# Xiaochaihutang Improves the Cortical Astrocyte Edema in Thioacetamide-Induced Rat Acute Hepatic Encephalopathy by Activating NRF2 Pathway

**DOI:** 10.3389/fphar.2020.00382

**Published:** 2020-04-16

**Authors:** Weiyi Jia, Jiajia Liu, Rui Hu, Anling Hu, Weiwei Tang, Lijuan Li, Jin Li

**Affiliations:** ^1^Key Laboratory of Infectious Disease and Biosafety, and Provincial Department of Education, Zunyi Medical University, Zunyi, China; ^2^Research Center for Medicine and Biology, Zunyi Medical University, Zunyi, China; ^3^Key Laboratory of Basic Pharmacology of Ministry of Education and Joint International Research Laboratory of Ethnocentric of Ministry of Education, Zunyi Medical University, Zunyi, China; ^4^Key Laboratory of Brain Science, Zunyi Medical University, Zunyi, China; ^5^Department of Pathophysiology, Basic Medical College, Zunyi Medical University, Zunyi, China

**Keywords:** Xiaochaihutang, acute hepatic encephalopathy, NRF2 pathway, brain edema, astrocyte edema

## Abstract

Oxidative stress induced by high ammonia, which leads to astrocyte edema, is the key to acute hepatic encephalopathy (AHE). Nuclear factor erythroid 2-related factor 2 (NRF2) has been implicated in oxidative stress, but the mechanism of NRF2 against ammonia-induced astrocytes edema has not been fully studied. We confirmed that the NRF2 pathway is related to brain edema caused by AHE and found that Xiaochaihutang (XCHT) could effectively activate the NRF2 pathway to treat AHE. The model of AHE was established with thioacetamide (TAA) in rats. Rat behaviors were observed, brain water content, blood ammonia levels, glutamine synthetase (GS), malondialdehyde (MDA), and total superoxide dismutase (T-SOD) were determined after XCHT treatment. Furthermore, the expression of NRF2 pathway proteins and mRNA, glial fibrillary acidic protein (GFAP) and aquaporins 4 (AQP4) were examined. In order to determine whether XCHT has a direct effect on cerebral edema caused by high ammonia, we examined the effect of XCHT compound serum on cortical astrocytes in the presence of ammonia, through microscopic observation and immunofluorescence (IF). Results showed that AHE induced by TAA changed the behavior of the rats, and increased brain water content, blood ammonia levels, GS and MDA content meanwhile decreasing T-SOD, but these symptoms were improved by treatment with XCHT. XCHT protected brain edema by activating the NRF2 pathway and increasing the expression of downstream proteins and genes. Astrocytes treated with 5 mM ammonia also showed an increase in the AQP4 protein expression but a decrease in XCHT compound serum and ammonia-induced cell edema groups. This study demonstrates that the NRF2 pathway is involved in the brain edema in AHE, and XCHT may represent a useful prescription for the treatment of AHE.

## Introduction

Hepatic Encephalopathy (HE) is a series of neuropsychological abnormalities secondary to liver dysfunction after excluding other known brain diseases, such as behavioral, intellectual and cognitive changes (Ferenci et al., 2002). Acute liver failure (ALF) is also called fulminant hepatic failure (FHF), which refers to various causes leading to severe acute liver injury, thus causing some potential and reversible diseases such as electrolyte metabolic disorder, jaundice, HE, etc (Trey and Davidson, 1970). Although the diseases are potentially reversible, they are all based on the loss of the detoxification function of the liver. The increase of toxins affects various systems in the body, and the mortality rate is high (Capocaccia and Angelico, 1991). One of the main components of ALF is the development of brain edema, which leads to increased intracranial pressure and brain herniation, eventually leading to death (Blei, 2010). The disease is also called AHE.

Evidence shows that brain edema in AHE is due to edema of astrocytes (Traber et al., 1987; Traber et al., 1989; Butterworth, 2003; Reinehr et al., 2010). An elevated ammonia level always exists in HE patients (Hazell and Butterworth, 1999). The occurrence of astrocyte edema seems to be affected by ammonia toxicity (Gregorios et al., 1985a; Gregorios et al., 1985b; Swain et al., 1992). Astrocytes mainly convert glutamic acid into glutamine through ammonia detoxification reaction, mediated by glutamine synthetase (Martinez et al., 1977). The dysfunction of astrocytes caused by ammonia-induced oxidative stress is also the core of AHE (Norenberg, 2003). GFAP is a landmark structural protein of astrocytes and participates in cell volume regulation. AQP4 specifically expresses on the capillary side of the protuberant foot plate of brain astrocytes and regulates the transmembrane flow of water. The changes in their expression are involved in astrocyte edema (Papadopoulos et al., 2002).

NRF2 is a major regulator in the redox balance (Cao et al., 2016). In normal conditions, NRF2 combines with Kelch-like ECH-associated protein1 (KEAP1) in an activity inhibition state (Tong et al., 2006). Under the action of the oxidative stress, NRF2 releases from KEAP1 and combines with the antioxidant response element (ARE) in the nucleus, then activates antioxidant enzyme genes expression such as heme oxygenase 1 (HO-1), NADPH quinine oxidoreductase 1 (NQO1), and GSH synthesis (GCLM, GCLC) (Kaspar et al., 2009; Liu et al., 2013). HO-1 is one of the most extensive antioxidant defense enzymes and a member of the heat shock protein family. It has anti-inflammatory, antioxidant, anti-apoptosis, and anti-proliferative effects. NQO1 is a flavin enzyme, which plays a key role in collective detoxification metabolism. GCLM and GCLC form glutamic acid cysteine ligase, which are two important genes in cell antioxidant defense mechanisms and play an important role in regulating glutathione synthesis. When cells are damaged by oxidative stress, gene expressions are up-regulated, glutathione synthesis is increased, and oxidative defense mechanism is enhanced (Liu et al., 2008; Reisman et al., 2009). It was found that NRF2 regulated the activity of the antioxidant enzymes and the expression of down-stream genes to protect against brain edema (Zhang et al., 2017; Chen et al., 2018; Duan et al., 2019). However, whether NRF2 plays a regulatory role in the occurrence and development of AHE needed further research and confirmation.

Xiaochaihutang (XCHT, Sho-Saiko-to, SST in Japan) is a classic formula of traditional Chinese medicine, which is first recorded in ‘Shang Han Lun’ and has the effects of anti-inflammation, hepatic protection, antipyretic and analgesic treatment. Our previous research found that XCHT can reduce liver cell necrosis and enhance liver function by activating the NRF2 pathway, thus playing a role in protecting the liver (Li et al., 2017; Hu et al., 2019; Jia et al., 2019). Moreover, studies have shown that XCHT can play a role in neurological diseases, such as the treatment of depression (Su et al., 2014), etc. However, whether XCHT treats brain edema through the NRF2 pathway in AHE rats remains to be clarified.

Therefore, this study aimed to investigate the pharmacological effects of XCHT on AHE rats, and subsequently delineated the underlying mechanisms which might be involved in it.

## Materials and Methods

### Animals, Drugs, and Treatments

Male Sprague-Dawley rats weighing 180-220 g (Experimental Animal Centre of the Third Military Medical University) were used for the experiments. Animals were fed in a room with a 12-h light/dark cycle at constant humidity and temperature (22-25°C) and had chow and water ad libitum placed in the cages. Animal maintenance and experimental protocols were carried out in accordance with guidelines approved by the Care Committee of Zunyi Medical University and the NIH guide of Humane Use.

Xiaochaihutang is a traditional Chinese Medicine. Xiaochaihu granule (containing Bupleurum falcatum L. (1.5 g), Scutellaria baicalensis Georgi (0.56 g), Codonopsis pilosula Franch (0.56 g), Pinellia temata Breitenbach (0.56 g), Glycyrrhiza glabra L.(0.56 g), Zingiber officinale Roscoe (0.56 g), and Zizyphus vulgaris Lam. (0.56 g)), referring to the ancient method of decocting, was purchased from Baiyunshan Guanghua Pharmaceutical Co., Ltd. (approval number K70079, Guangzhou, China). According to the 2015 edition of the Pharmacopeia Committee of China (Pharmacopeia Committee of China, 2015), baicalin is used as the index of Xiaochaihu granule, and the content of baicalin in Xiaochaihu granule, measured by high performance liquid chromatography (HPLC) in our previous studies, is 24.307 mg/10g. This conforms to the requirement of not less than 20 mg/10g.

After acclimatization for one week, rats were randomly divided into five groups (50, n = 10): vehicle group, model group, XCHT treatment groups (the instruction of Xiaochaihu granule suggests that patients should take 6-12 g/day, and considering the dose conversion from human to rats, the dose of XCHT is clinically relevant: 1.0 g/kg, 2.0 g/kg, 4.0 g/kg). To induce acute hepatic encephalopathy, rats in model and XCHT groups were given TAA (300 mg/kg/day, i.p.) for three consecutive days (Jayakumar et al., 2014). Starting from the 4^th^ day, the animals in treatment groups were given the appropriate doses of XCHT granules for 2 weeks (1 mL/100 g/day, i.g.). In the end after 2 weeks of treatment, the mortality rate of rats was about 10%. Then behavior was tested and all rats were sacrificed to obtain blood and cerebral cortex samples.

### Observation and Measurement of Rat Behaviors

Rats were observed after administrations and recorded in an open field. Behaviors included mortality, body weight and auto-activity. All studies were performed under strictly standardized conditions in a dark room for 10 min. The total distances were recorded to reflect the auto-activities of rats with AHE. The activities were defined as zero in dead rats (Wang et al., 2017).

### Brain Water Measurement

Brain water content was evaluated as previously described (Duris et al., 2011). The cerebral cortex was removed and separated into 2 parts. The left hemisphere was weighed for wet weight. Tissue dried for 72 h in an oven at 110°C, and dry weights were determined. The percentage of water content was calculated as [(wet weight − dry weight)/wet weight] × 100%.

### Biochemical Analysis

The plasma was separated and the blood ammonia level was detected according to the instructions of the kit (Nanjing Jiancheng Bioengineering Institute, Nanjing, China). The cortex was isolated from the brain of rat. After homogenizing the cortex, the levels of GS, MDA and T-SOD were determined according to the instructions of the kit (Nanjing Jiancheng Bioengineering Institute, Nanjing, China). Protein concentrations were determined using the Bradford method.

### IHC of GFAP in AHE

IHC for GFAP was carried out on cerebral cortex tissue slices after deparaffinization, rehydration, antigen unmasking by heat treatment, and 5% normal goat serum blocking. Slices were incubated with a specific primary antibody overnight at 4 °C, then processed using a GTVision kit (Gene Tech, Shanghai, China) according to the technical manual. Five areas of each slice were randomly examined under an optical microscope (OLYMPUS, BX5, Japan).

### Astrocyte Cultures

Primary cultures of cortical astrocytes were prepared as described previously (Guo et al., 2014). Primary cultures of cortical astrocytes were prepared from 1/2-day-old rats. Briefly, the cerebral cortex was stripped from the meninges, minced and dissociated by trituration, passed through sterile nylon sieves and then placed in Dulbecco's modified Eagle medium (DMEM) containing 10% FBS. The medium was changed after 24 h. Astrocytes were incubated at 37 °C in a humidified chamber provided with 5% CO_2_ and 95% air. Cultures consisted of at least 95% astrocytes and were determined by GFAP IF.

There were three groups: 10% (v/v) control-serum group (vehicle group), 10% (v/v) control-serum and NH_4_Cl (5 nM) group (model group), and 10% (v/v) XCHT compound serum and NH_4_Cl (5 nM) group (XCHT group). Serums were provided by preliminary work of our group (Hu et al., 2019). In brief, 30 rats were randomly divided into two equal groups, which includes the control (vehicle) group (n=15) and XCHT treatment group (n=15). The XCHT group received an oral administration of 2 g/kg decoction for 6 days (considering the dose conversion from human to rat, the dose of XCHT was clinically relevant; according to the results of previous in vivo experiments, we chose 2.0 g/kg for administration). A distilled water vehicle control was given to the control group. Then the blood samples were obtained from the abdominal aorta 2 h after oral administration and centrifuged at 3500 rpm for 15 min. The serum samples were mixed and inactivated.

### IF Microscopy

Astrocytes were cultured on glass coverslips for 24 h and treated for a further 24 h. Coverslips were washed with PBS, fixed in 4% (v/v) paraformaldehyde for 20 min at room temperature, washed prior to detergent extraction with 0.5% (v/v) Triton X-100 for 20 min., then saturated with PBS containing 5% (v/v) normal goat serum for 30 min. Next, cells were incubated with the specific primary antibody for AQP4 over-night, washed, then incubated with secondary antibody for 1 h at 37 °C. Finally, cells were stained for 5 min with 4,6-diamidino-2-phenylindole (DAPI). OLYMPUS IX73 microscope was used to observe the slides.

### Real Time PCR Analysis

Total RNA was extracted from cerebral cortex tissues and treated cortical astrocytes using RNAiso plus (TAKARA, Dalian, China). The primers were designed and synthesized by TAKARA Systems. The sequences of the primers were listed in **Table 1**. The total RNA was reverse-transcribed with 5 × PrimeScripe TM Buffer, Prime Script TMRT Enzyme Mix, and Oligo dT Primer. The SYBR green PCR Master Mix was chosen for real time PCR analysis. A relative quantitative method was used for analysis of results.

**Table 1:** The primer sequence for RT-PCR.

Gene	Sequence (5′~3′)	
	Forward primer	Reverse primer
β-actin	GGAGATTACTGCCCTGGCTCCTA	GACTCATCGTACTCCTGCTTGCTG
NRF2	TTGGCAGAGACATTCCCATTTGTA	GAGCTATCGAGTGACTGAGCCTGA
KEAP1	CATCGGCATCGCCAACTTC	GCTGGCAGTGTGACAGGTTGA
HO-1	AGGTGCACATCCGTGCAGAG	CTTCCAGGGCCGTATAGATATGGTA
NQO1	TGGAAGCTGCAGACCTGGTG	CCCTTGTCATACATGGTGiGCATAC
GCLC	CTGCACATCTACCACGCAGTCA	ATCGCCGCCATTCAGTAACAA
GCLM	AGACCGGGAACCTGCTCAAC	GATTTGGGAGCTCCATTCATTCA
GFAP	TCGTGTGGATCTGGAGAGGAAGG	AGAGCCGCTGTGAGGTCTGG
AQP4	GAAGGCGGTCACAGCAGAGTTC	TGATGTGGCCGAAGCACTGAAC

### Western Blot Analysis

Cytoplasmic and nuclear protein of cerebral cortex tissues and treated cortical astrocytes were extracted by NE-PER Nuclear and Cytoplasmic Extraction Reagents (Thermo-Scientific, Rockford, United States). Equal amounts of protein (15 μg) were separated on 10% SDS-polyacrylamide gel. The blots were incubated overnight with individual protein anti-bodies (1:1000, Abcam, Cambridge, UK), washed, incubated with anti-rabbit IgG, anti-mouse IgG (1:5000, ZSGB-BIO, Beijing, China) for 2 h, and developed by ECL western blotting substrate. Western blot signals were quantified by Imager (Bio-Rad, America).

### Statistical Analysis

All data were expressed as mean ± standard deviation (S.D). Statistical analysis was performed with Student’s *t* test and ANOVA with the Tukey post hoc test was used if more than two experimental groups were compared, and *p* < 0.05 was considered statistically significant. Data were analyzed with GraphPad Prism 5.0 (La Jolla, CA, USA).

## Results

### XCHT Treatment Suppressed the Development of TAA-Induced AHE

**Figure 1:**
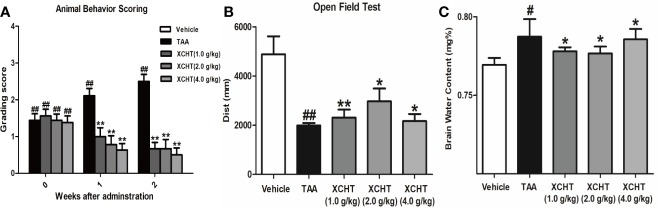
Acute hepatic encephalopathy (AHE) was induces by repeated injection of 300 mg/kg/day thioacetamide (TAA) for 3 days (1 mL/kg in saline, i.p.). From the 4th day, the animals in treatment groups were given the XCHT granules (1.0 g/kg, 2.0 g/kg and 4.0 g/kg, 1mL/100 g/day, i.g.) for 2 weeks. **(A, B)** Measurement of the rat behaviors [**(A)** Animal behavior scoring. **(B)** Open field test. Rats in each group were evaluated for total distance traveled]. **(C)** Brain water content ([(wet weight – dry weight)/wet weight] × 100%). Data were expressed as mean ± SD (n = 5-7). ^#^*p* < 0.05, ^##^*p*< 0.01 vs. the vehicle group. *p < 0.05, **p < 0.01 vs. the TAA group.

**Figure 2:**
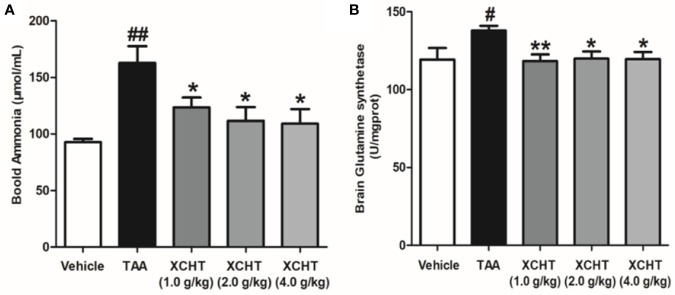
Effect of XCHT on ammonia level in TAA induced AHE rats. **(A)** Ammonia plasma levels in all groups of rats. **(B)** GS levels in cerebral cortex of rats. Data were expressed as mean ± SD (n = 5-7). ^#^p < 0.05, ^##^p < 0.01 vs. the vehicle group. *p < 0.05, **p < 0.01 vs. the TAA group.

To analyze the effect of XCHT on TAA-induced AHE in rats, the degree of liver injury in the rats was at first measured (**Supplementary Figure 1**). Subsequently the behaviors [scoring (**Figure 1A**), total distance traveled (**Figure 1B**)], the brain cortical water content (**Figure 1C**), the ammonia plasma (**Figure 2A**), and the GS (**Figure 2B**) levels in all groups of rats were measured. Compared with the vehicle group, significant changes in behavior, decreased auto-action, increased brain water content, blood ammonia and GS in the cerebral cortex were observed in the TAA group relative to the vehicle group. Whereas in rats treated with XCHT behavior scoring improved during the first week, and increased automatic action, decreased brain water content, blood ammonia, and GS were observed. Together, the data presented here demonstrated that XCHT enabled the healing of TAA-induced AHE.

### XCHT Reduced the Degree of TAA-induced Oxidative Damage in AHE Rats

**Figure 3:**
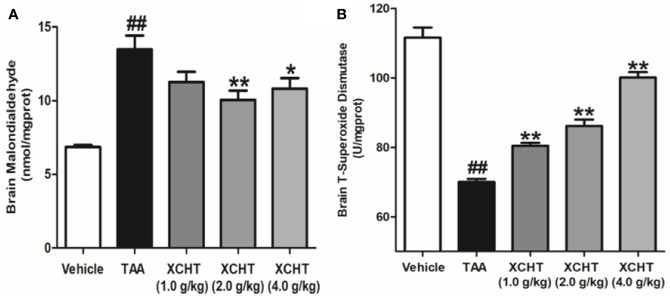
Effects of different doses of XCHT on oxidative stress level [MDA **(A)** and T-SOD **(B)**] in cerebral cortex of TAA induced AHE rats. Data were expressed as mean ± SD (n = 5-7). ^##^p < 0.01 vs. the vehicle group. *p < 0.05, **p < 0.01 vs. the TAA group.

In order to detect the oxidative damage degree of AHE rat, we measured the levels of MDA (**Figure 3A**) and T-SOD (**Figure 3B**) in the cerebral cortex by colorimetry. Compared with the vehicle group, MDA level was found to be dramatically increased in the TAA group, and in contrast the level of T-SOD was obviously decreased. However, the groups of XCHT treatment noticeably prevented oxidative stress production in a dose-dependent manner. The data presented here demonstrated that XCHT enabled the healing of TAA-induced AHE by reducing oxidative stress damage.

### XCHT Activated NRF2 Pathway in AHE Rats

**Figure 4:**
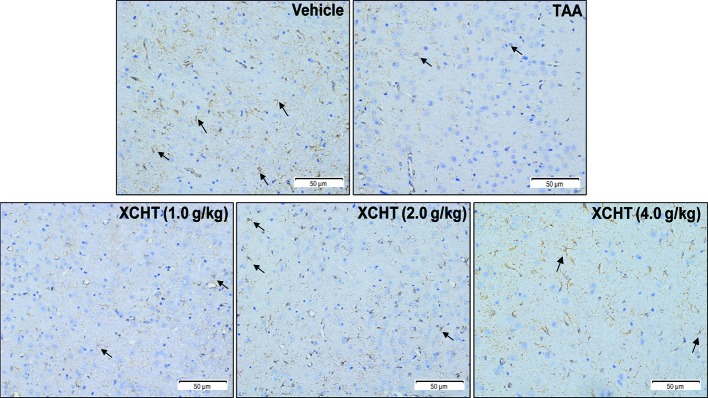
Effect of XCHT on structure of the astrocytes in cerebral cortex of AHE rats. GFAP expression in cerebral cortex of rat brain (IHC, 200×). The decrease of GFAP immunostaining in cortex of TAA group rats compared to the vehicle group, which indicated that the structure of astrocytes was destroyed and brain edema occurred. In contrast, the expression of GFAP in astrocytes increased in the treatment groups with different doses.

**Figure 5:**
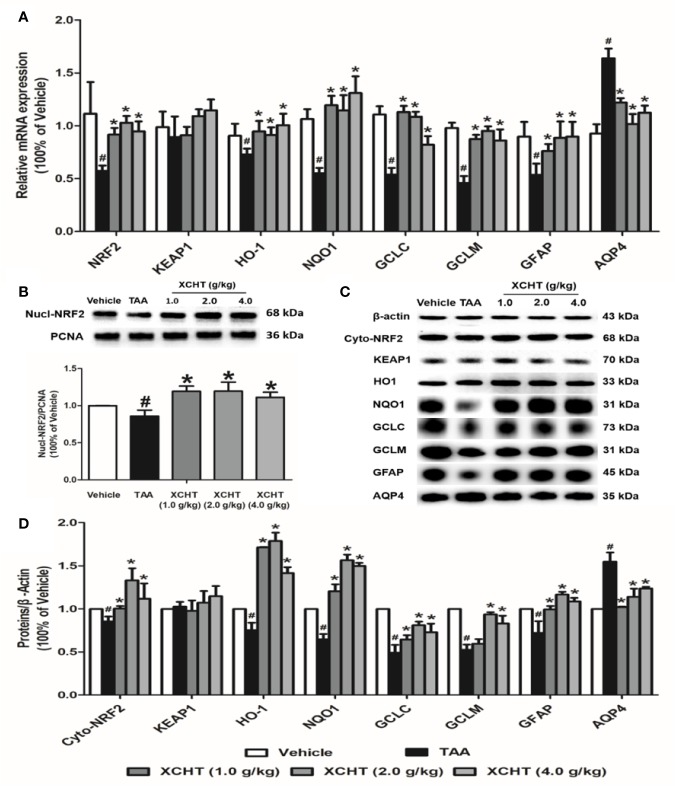
Effect of XCHT on TAA-induced AHE in rats by activating cortical astrocytes NRF2 pathway. **(A)** The mRNA expression of NRF2, KEAP-1, HO-1, NQO-1, GCLC, GCLM, GFAP and AQP4. **(B)** The protein expression of Nucl-NRF2. **(C, D)** The protein expression of Cyto-NRF2, KEAP-1, HO-1, NQO-1, GCLC, GCLM, GFAP and AQP4. Data were expressed as mean ± SD (n = 5-7). ^#^p < 0.05, *p < 0.01 vs. the TAA group.

To examine whether different doses of XCHT inhibit the destruction of cortical astrocytes in an experimental model of AHE (TAA treated rats), cortical sections from each group (n=3) were immunostained with GFAP, and images were captured with a microscope. Faint GFAP staining of astrocytes were observed in the TAA group. However, intense GFAP IHC was found in XCHT treatment groups (**Figure 4**). In order to determine whether XCHT could protect the cerebral cortex by activating the NRF2 pathway, the expression of NRF2 pathway proteins and genes (Nucl-NRF2 (**Figure 5B**), Cyto-NRF2, KEAP1, HO-1, NQO1, GCLC, GCLM) (**Figures 5A, C**) were further quantified in each group by western blots and real time PCR. Compared with the vehicle group, the TAA group showed a decrease in Nucl-NRF2 and proteins on the NRF2 pathway. In contrast, XCHT treatment groups showed an increase in Nucl-NRF2 and proteins on the NRF2 pathway. At the same time, the level of GFAP and AQP4 in the cerebral cortex was detected and compared with the model group. The expression of GFAP in the XCHT treatment group was increased while there was a decrease in AQP4 protein (**Figures 5A, C**). These results indicated that XCHT might activate the NRF2 pathway to improve brain edema induced by AHE in rats.

### Effect of XCHT Compound Serum on the Ammonia-Induced Cell Edema in Astrocytes

**Figure 6:**
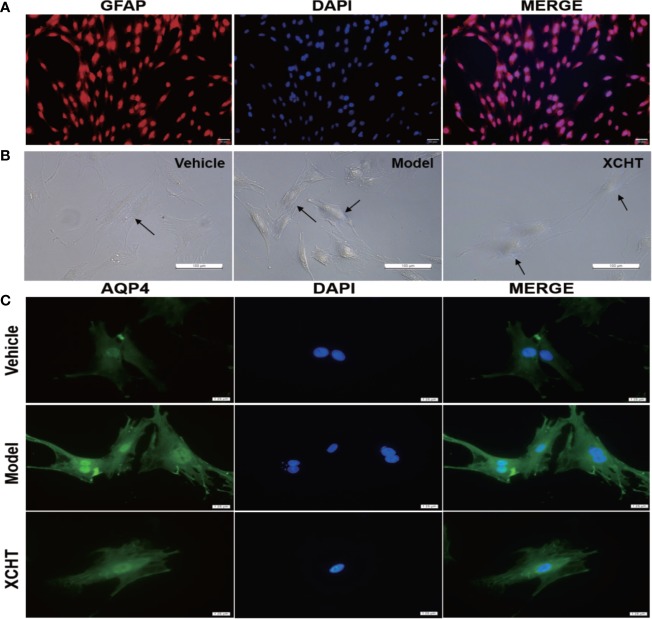
Effect of XCHT compound serum on ammonia-induced astrocyte swelling. **(A)** Purity of primary astrocyte culture. Immunolabeling of primary rat astrocyte cultures with the markers GFAP (Red) revealed pure primary astrocyte culture. Nucleus were stained with 4′,6′-diamidino-2-phenylindole (DAPI) (Blue). (Staining, 100×) **(B)** Morphological overview of astrocyte culture after treatment. **(C)** Immunolabeling of primary rat astrocyte cultures with the markers AQP4 (Green), Nucleus were stained with 4′,6′-diamidino-2-phenylindole (DAPI) (Blue).

The obtained astrocyte culture purity and maturity was put under immunocytochemical examination using an antibody against to the GFAP protein. The astrocyte purity was greater than 95% for the following experiments (**Figure 6A**).

Since XCHT has a protective effect on the liver and reduces the ammonia level in the body, whether or not XCHT has a direct therapeutic effect on cerebral edema caused by AHE needs to be further determined. Cultures were divided between the vehicle group (10% control-serum), the model group (5 mM NH_4_Cl and 10% control-serum) and the XCHT group (5 mM NH_4_Cl and 10% XCHT compound serum). After 24 h treatment, the cell morphology was observed by a microscope. An increased tense had been seen in cell volume and the cell refractive index was observed after exposure to ammonia for 24 h. Such edema was attenuated by treatment of cells with XCHT compound serum (**Figure 6B**). Compared with the vehicle group, intense AQP4 IF cortical astrocytes were found in the model group. But faint AQP4 staining of astrocytes were observed in the XCHT group (**Figure 6C**).

## Discussion

In our present study, we investigated whether XCHT has a potentially protective effect on brain edema associated with AHE, which works by activating the NRF2 pathway. To clarify the effect of XCHT on brain edema caused by AHE, we performed in vivo and vitro experiments respectively and detected the expression of GFAP, AQP4 involved in astrocyte edema as well as the NRF2 pathway (Nucl-NRF2, Cyto-NRF2, HO-1, NQO-1, GCLC and GCLM). This study demonstrates for the first time that the occurrence of AHE-related brain edema may be associated with the NRF2 pathway, and that XCHT, by offering a protective role against ammonia-induced brain/astrocyte edema through activating the NRF2 pathway, may be a promising candidate for AHE treatment.

As mentioned earlier, studies demonstrated that the occurrence of brain edema caused by AHE was related to oxidative stress (Bodega et al., 2015; Mladenović et al., 2015). Since it is the central regulator of cell antioxidant response, the NRF2 pathway plays an important role in the mechanism of cell resistance to internal and external oxidative stress. Studies elaborated that the NRF2 pathway was involved in brain edema caused by various factors (Yang et al., 2016; Gao et al., 2018; Jin et al., 2018). Although some studies analyzed that AHE-induced astrocyte edema was related to the downstream gene HO-1 of the NRF2 pathway (Oenarto et al., 2016), there is no evidence that it is directly related to NRF2 pathway. Therefore, we assumed that the occurrence of brain edema caused by AHE may be related to the NRF2 pathway. In vivo and vitro, the results showed that ammonia inhibited and reduced the activation of the NRF2 pathway in astrocytes, thus leading to the occurrence of brain edema (**Figures 4**, **6**), which is consistent with our hypothesis.

XCHT plays a protective role in disease models damaged by oxidative stress. Our previous studies proved that XCHT could reduce the related symptoms of liver fibrosis and ALF in rats by activating the NRF2 pathway (Jia et al., 2019). Wang et al. proved that XCHT could protect human skin fibroblasts from oxidative stress injury induced by hydrogen peroxide through blocking NF-κB (Wang et al., 2018). At present, studies showed that XCHT could be able to treat neurological related diseases, such as depression (Ma et al., 2017). Therefore, we assumed that XCHT could also activate the NRF2 pathway in the brain after AHE, and reduce the occurrence of brain edema by inhibiting oxidative stress. For this reason, XCHT was given to treat AHE rats in this study. The results showed that XCHT activated the NRF2 pathway and promoted NRF2 translocation from cytoplasm to the nucleus. This translocation alleviates the symptoms of brain edema caused by oxidative stress injury and also alleviates the occurrence of AHE. However, the effect of XCHT treatment with 1.0 or 4.0 mg/kg on rats was not as good as that with 2.0 mg/kg (**Figure 5**). Although the cause of this effect is not clear, low or high concentrations of XCHT may produce non-specific effects.

Previously, it was proved that administration of XCHT to ALF rats could improve liver oxidative damage through the NRF2 pathway, which indicated that XCHT had beneficial effects on ALF (Jia et al., 2019). As XCHT is reported to have a hepato-protective effect, the beneficial effect of XCHT on AHE-induced brain edema observed in this study might be because of the secondary one liver function improvement. And we further verified this conjecture.

In order to simulate the environment in vivo, we selected 10% XCHT compound serum to treat astrocytes in ammonia environment according to previous studies. Studies found that XCHT compound serum contained a variety of active ingredients (such as liquiritin, rutin, zingerone, baicalin, quercetin, etc.) (Sun et al., 2015; Chen et al., 2017). We also detected baicalin in the serum used in this study (**Supplementary Figure 2**), and previous studies found that liquiritin, rutin, baicalin and other components could enter the brain through the blood-brain barrier (BBB) (Huang et al., 2015; Chen et al., 2017; Enogieru et al., 2018). These results showed that XCHT compound serum could protect the structure of astrocytes and reduce AQP4 expression in cells. These results were consistent with our previous work in vitro. All the results proved that the recovery from the brain edema in TAA-induced AHE rats after treatment with XCHT might be due to a direct effect of XCHT on brain edema and a direct effect of improvement in liver function.

However, the study had several limitations. Firstly, since XCHT is a compound Chinese Medicine, which has multitarget features and a variety of protective effects such as antioxidative and anti-inflammatory properties (Hu et al., 2019), we should consider if its protection in AHE is related to its anti-inflammation or other properties in our study. Secondly, in order to simulate the internal environment, we should choose drug-containing cerebrospinal fluid for the cell part. However, considering the small amount of cerebrospinal fluid in rats and the large amount needed in the experimental process, we chose drug-containing serum for this study. Although it was proven that baicalin produced after XCHT entering the body for metabolism could enter the brain, the effect of serum on astrocytes may cause slight deviation in the experimental results. Thirdly, our experiment treated XCHT only at the beginning of AHE. The therapeutic effect of XCHT on AHE in different clinical stages needed further study.

In summary, NRF2 pathway protein and mRNA levels were decreased in the cerebral cortex in the TAA model of AHE rats, but treatment with XCHT reduced the brain edema as well as improved their clinical status. Decreased NRF2 was associated with brain edema and activating this pathway with XCHT significantly reduced the edema. Astrocytes treated with ammonia showed the same change in AQP4 as the TAA model of AHE. Our findings suggest that the NRF2 pathway is related to brain/astrocyte edema in AHE, and XCHT might be an effective method for the treatment of brain edema associated with AHE.

## Data Availability Statement

The raw data supporting the conclusions of this manuscript will be made available by the authors, without undue reservation, to any qualified researcher.

## Ethics Statement

Animal maintenance and experimental protocols were carried out in accordance with guidelines approved by the Care Committee of Zunyi Medical University and the NIH guide of Humane Use.

## Author Contributions

All authors contributed to the study conception and design. JL and WJ participated in research design. Material preparation, data collection and analysis and the manuscript were performed by WJ, JJL, and RH. AH, WT, and LL performed the data analysis. All authors commented on previous versions of the manuscript. All authors read and approved the final manuscript.

## Funding

This work was partly supported by the National Natural Science Foundation Committee (NSFC) of China (81360661 and 81560592).

## Conflict of Interest

The authors declare that the research was conducted in the absence of any commercial or financial relationships that could be construed as a potential conflict of interest.

## References

[B1] Blei A. T. (2010). Cerebral edema and intracranial hypertension in acute liver failure: distinct aspects of the same problem. Hepatology 13, 376–379. 10.1002/hep.1840130227 1847353

[B2] Bodega G. Segura B. Ciordia S. Mena M. C. López-Fernández L. A. García M. I. (2015). Ammonia affects astroglial proliferation in culture. PloS One 10, e0139619. 10.1371/journal.pone.0139619 26421615 PMC4589356

[B3] Butterworth R. F. (2003). Pathogenesis of hepatic encephalopathy: new insights from neuroimaging and molecular studies. J. Hepatol. 39, 278–285. 10.1016/S0168-8278(03)00267-8 12873828

[B4] Cao L.-J. Li H.-D. Yan M. Li Z.-H. Gong H. Jiang P. (2016). The protective effects of isoliquiritigenin and glycyrrhetinic acid against triptolide-induced oxidative stress in HepG2 cells involve Nrf2 activation. Evid. Based. Complement. Alternat. Med. 2016, 8912184. 10.1155/2016/8912184 26904149 PMC4745288

[B5] Capocaccia L. Angelico M. (1991). Fulminant hepatic failure. Clinical features, etiology, epidemiology, and current management. Digest. Dis. Sci. 36, 775–779. 10.1007/BF01311236 2032520

[B6] Chen T.-F. Liu J.-X. Zhang Y. Lin L. Song W.-T. Yao M.-J. (2017). Analysis on microdialysis probe recovery of baicalin in vitro and in vivo based on LC-MS /MS. Chin. J. Chin. Mater. Med. 42, 2168–2174. 10.19540/j.cnki.cjcmm.20170307.001 28822164

[B7] Chen H.-Y. Cao J. Zhu Z.-Y. Zhang G.-X. Shan L.-C. Yu P. (2018). A novel tetramethylpyrazine derivative protects against glutamate-induced cytotoxicity through PGC1α/Nrf2 and PI3K/Akt signaling pathways. Front. Neurosci. 12, 567. 10.3389/fnins.2018.00567 30158850 PMC6104130

[B8] Duan J.-L. Cui J. Yang Z.-F. Guo C. Cao J.-Y. Xi M.-M. (2019). Neuroprotective effect of Apelin 13 on ischemic stroke by activating AMPK/GSK-3β/Nrf2 signaling. J. Neuroinflamm. 16, 24. 10.1186/s12974-019-1406-7 PMC6357442 30709405

[B9] Duris K. Manaenko A. Suzuki H. Rolland W. Tang J. Zhang J. H. (2011). Sampling of CSF via the cisterna magna and blood collection via the heart affects brain water content in a rat SAH model. Transl. Stroke Res. 2, 232–237. 10.1007/s12975-010-0063-z 21666823 PMC3109988

[B10] Enogieru A. B. Haylett W. Hiss D. C. Bardien S. Ekpo O. E. (2018). Rutin as a Potent Antioxidant: Implications for Neurodegenerative Disorders. Oxid. Med. Cell. Longev. 27, 6241017. 10.1155/2018/6241017 PMC6040293 30050657

[B11] Ferenci P. Lockwood A. Mullen K. Tarter R. Weissenborn K. Blei A. T. (2002). Hepatic encephalopathy-Definition, nomenclature, diagnosis, and quantification: Final report of the Working Party at the 11^th^ World Congresses of Gastroenterology, Vienn. Hepatology 35, 716–721. 10.1053/jhep.2002.31250 11870389

[B12] Gao Y. Fu R.-R. Wang J. Yang X. Wen L. Feng J. (2018). Resveratrol mitigates the oxidative stress mediated by hypoxic-ischemic brain injury in neonatal rats via Nrf2/HO-1 pathway. Pharm. Biol. 56, 440–449. 10.1080/13880209.2018.1502326 30460866 PMC6249550

[B13] Gregorios J. B. Mozes L. W. Norenberg L. O. Norenberg M. D. (1985a). Morphologic effects of ammonia on primary astrocyte cultures. I. Light microscopic studies. J. Neuropathol. Exp. Neurol. 44, 397–403. 10.1097/00005072-198507000-00003 4009208

[B14] Gregorios J. B. Mozes L. W. Norenberg M. D. (1985b). Morphologic effects of ammonia on primary astrocyte cultures. II. Electron microscopic studies. J. Neuropathol. Exp. Neurol. 44, 404–414. 10.1097/00005072-198507000-00004 4040156

[B15] Guo H. Mao M. Yu D. Zhou H. Tong Y. (2014). A modified culture method for astrocytes from rat cortical tissue in vitro. Zhongguo. Dang. Dai. Er. Ke. Za. Zhi. 16, 1271–1274. 10.7499/j.issn.1008-8830.2014.12.017 25523579

[B16] Hazell A. S. Butterworth R. F. (1999). Hepatic encephalopathy: an update of pathophysiologic mechanisms. Exp. Biol. Med. 222, 99–112. 10.1046/j.1525-1373.1999.d01-120.x 10564534

[B17] Hu R. Jia W.-Y. Xu S.-F. Zhu Z.-W. Xiao Z. Yu S.-Y. (2019). Xiaochaihutang inhibits the activation of hepatic stellate cell line T6 through the Nrf2 pathway. Front. Pharmacol. 9, 1516. 10.3389/fphar.2018.01516 30666206 PMC6330344

[B18] Huang X. Wang Y. Ren K. (2015). Protective Effects of Liquiritin on the Brain of Rats with Alzheimer’s Disease. West. Indian Med. J. 64, 468–472. 10.7727/wimj.2016.058 27399208 PMC4961333

[B19] Jayakumar A. R. Valdes V. Tong X. Y. Shamaladevi N. Gonzalez W. Norenberg M. D. (2014). Sulfonylurea receptor 1 contributes to the astrocyte swelling and brain edema in acute liver failure. Transl. Stroke Res. 5, 28–37. 10.1007/s12975-014-0328-z 24443056 PMC4714761

[B20] Jia W.-Y. Liu J.-J. Hu R. Hu A.-L. Xu S.-F. Wang H. (2019). Therapeutic mechanism of xiaochaihu granule on acute liver injury induced by thioacetamide in rats through Nrf2 pathway. Chin. J. Exp. Tradit. Med. Form. 25, 54–59. 10.13422/j.cnki.syfjx.20190840

[B21] Jin X.-X. Liao Y.-J. Tan X.-Q. Wang G. Zhao F. Jin Y. (2018). Involvement of CYP2E1 in the course of brain edema induced by subacute poisoning with 1,2-Dichloroethane in mice. Front. Pharmacol. 9, 1317. 10.3389/fphar.2018.01317 30524279 PMC6262393

[B22] Kaspar J. W. Niture S. K. Jaiswal A. K. (2009). Nrf2: INrf2 (Keap1) signaling in oxidative stress. Free. Radic. Biol. Med. 47, 1304–1309. 10.1016/j.freeradbiomed.2009.07.035 19666107 PMC2763938

[B23] Li J. Hu R. Xu S.-F. Li Y. Qin Y. Wu Q. (2017). Xiaochaihutang attenuates liver fibrosis by activation of Nrf2 pathway in rats. Biomed. Pharmacother. 96, 847–853. 10.1016/j.biopha.2017.10.065 29078262

[B24] Liu J. Wu Q. Lu Y.-F. Pi J. (2008). New insights into generalized hepatoprotective effects of oleanolic acid: key roles of metallothionein and Nrf2 induction. Biochem. Pharmacol. 76, 922–928. 10.1016/j.bcp.2008.07.021 18706400

[B25] Liu J. Wu K.-C. Lu Y.-F. Ekuase E. Klaassen C. D. (2013). NRF2 protection against liver injury produced by various hepatotoxicants. Oxid. Med. Cell. Longev. 2013, 305861. 10.1155/2013/305861 23766851 PMC3676920

[B26] Ma J. Wang F. Yang J. Dong Y. Su G. Zhang K. (2017). Xiaochaihutang attenuates depressive/anxiety-like behaviors of social isolation-reared mice by regulating monoaminergic system, neurogenesis and BDNF expression. J. Ethnopharmacol. 208, 94–104. 10.1016/j.jep.2017.07.005 28687505

[B27] Martinez H. A. Bell K. P. Norenberg M. D. (1977). Glutamine synthetase: glial localization in brain. Science 195, 1356–1358. 10.1126/science.14400 14400

[B28] Mladenović D. Petronijević N. Stojković T. Velimirović M. Jevtić G. Hrnčić D. (2015). Finasteride has regionally different effects on brain oxidative stress and acetylcholinesterase activity in acute thioacetamide-induced hepatic encephalopathy in rats. Plos. One 10, e0134434. 10.1371/journal.pone.0134434 26241899 PMC4524603

[B29] Norenberg M. D. (2003). Oxidative and nitrosative stress in ammonia neurotoxicity. Hepatology 37, 245–248. 10.1053/jhep.2003.50087 12540772

[B30] Oenarto J. Karababa A. Castoldi M. Bidmon H. J. Görg B. Häussinger D. (2016). Ammonia-induced miRNA expression changes in cultured rat astrocytes. Sci. Rep. 6, 18493. 10.1038/srep18493 26755400 PMC4709596

[B31] Papadopoulos M. C. Krishna S. Verkman A. S. (2002). Aquaporin water channels and brain edema. Mt. Sinai. J. Med. 69, 242–248. 10.1097/01.md.0000032520.993581e 12357265

[B32] Pharmacopeia Committee of China (2015) (Beijing: Chinese Medical Press), 576–577.

[B33] Reinehr R. Görg B. Becker S. Qvartskhava N. Bidmon H. J. Selbach O. (2010). Hypoosmotic swelling and ammonia increase oxidative stress by NADPH oxidase in cultured astrocytes and vital brain slices. Glia 55, 758–771. 10.1002/glia.20504 17352382

[B34] Reisman S. A. Aleksunes L. M. Klaassen C. D. (2009). Oleanolic acid activates Nrf2 and protects from acetaminophen hepatotoxicity via Nrf2-dependent and Nrf2-independent processes. Biochem. Pharmacol. 77, 1273–1282. 10.1016/j.bcp.2008.12.028 19283895 PMC2745914

[B35] Su G.-Y. Yang J.-Y. Wang F. Xiong Z.-L. Hou Y. Zhang K. (2014). Xiaochaihutang prevents depressive-like behaviour in rodents by enhancing the serotonergic system. J. Pharm. Pharmacol. 66, 823–834. 10.1111/jphp.12201 24359306

[B36] Sun R. Zeng M. Du T. Li L. Yang G. Hu M. (2015). Simultaneous determinations of 17 marker compounds in Xiao-Chai-Hu-Tang by LC-MS/MS: Application to its pharmacokinetic studies in mice. J. Chromatogr. B. Analyt. Technol. Biomed. Life Sci. 15, 12–21. 10.1016/j.jchromb.2015.09.004 PMC4608253 26397748

[B37] Swain M. Butterworth R. F. Blei A. T. (1992). Ammonia and related amino acids in the pathogenesis of brain edema in acute ischemic liver failure in rats. Hepatology 15, 449–453. 10.1002/hep.1840150316 1544626

[B38] Tong K. I. Kobayashi A. Katsuoka F. Yamamoto M. (2006). Two-site substrate recognition model for the Keap1-Nrf2 system: a hinge and latch mechanism. Biol. Chem. 387, 1311–1320. 10.1515/BC.2006.164 17081101

[B39] Traber P. G. Canto M. D. Ganger D. R. Blei A. T. (1987). Electron microscopic evaluation of brain edema in rabbits with galactosamine-induced fulminant hepatic failure: Ultrastructure and integrity of the blood-brain barrier. Hepatology 7, 1272–1277. 10.1002/hep.1840070616 3679092

[B40] Traber P. Dalcanto M. Ganger D. Blei A. T. (1989). Effect of body temperature on brain edema and encephalopathy in the rat after hepatic devascularization. Gastroenterology 96, 885–891. 10.1016/S0016-5085(89)80092-7 2914649

[B41] Trey C. Davidson C. S. (1970). The management of fulminant hepatic failure. Prog. Liver Dis. 3, 282–298. 10.1007/BF01738610 4908702

[B42] Wang W.-W. Zhang Y. Huang X.-B. You N. Zheng L. Li J. (2017). Fecal microbiota transplantation prevents hepatic encephalopathy in rats with carbon tetrachloride-induced acute hepatic dysfunction. World J. Gastroenterol. 23, 6983–6994. 10.3748/wjg.v23.i38.6983 29097871 PMC5658316

[B43] Wang C.-Y. Jiang M. Hou G.-Y. Hou J. G. (2018). Saikokeishito Protecting HumanDermal Fibroblast Cell from Oxidative Damage Induced by H_2_O_2_. Chin. J. Lab. Diagn. 22, 1074–1077. 10.3969/j.issn.1007-4287.2018.06.044

[B44] Yang Y.-Q. Wang H.-D. Li L.-W. Li X. Wang Q. Ding H. (2016). Sinomenine provides neuroprotection in model of traumatic brain injury via the Nrf2-ARE pathway. Front. Neurosci. 10, 580. 10.3389/fnins.2016.00580 28066165 PMC5179594

[B45] Zhang L. Wang H. Fan Y. Gao Y. Li X. Hu Z. (2017). Fucoxanthin provides neuroprotection in models of traumatic brain injury via the Nrf2-ARE and Nrf2-autophagy pathways. Sci. Rep. 7, 46763. 10.1038/srep46763 28429775 PMC5399453

