# Supplementary figures and images for: Xiaochaihutang Improves the Cortical Astrocyte Edema in Thioacetamide-Induced Rat Acute Hepatic Encephalopathy by Activating NRF2 Pathway
Source: Front Pharmacol. 2020 Apr 16;11:382. doi: 10.3389/fphar.2020.00382 (PMC7179068; doi:10.3389/fphar.2020.00382)

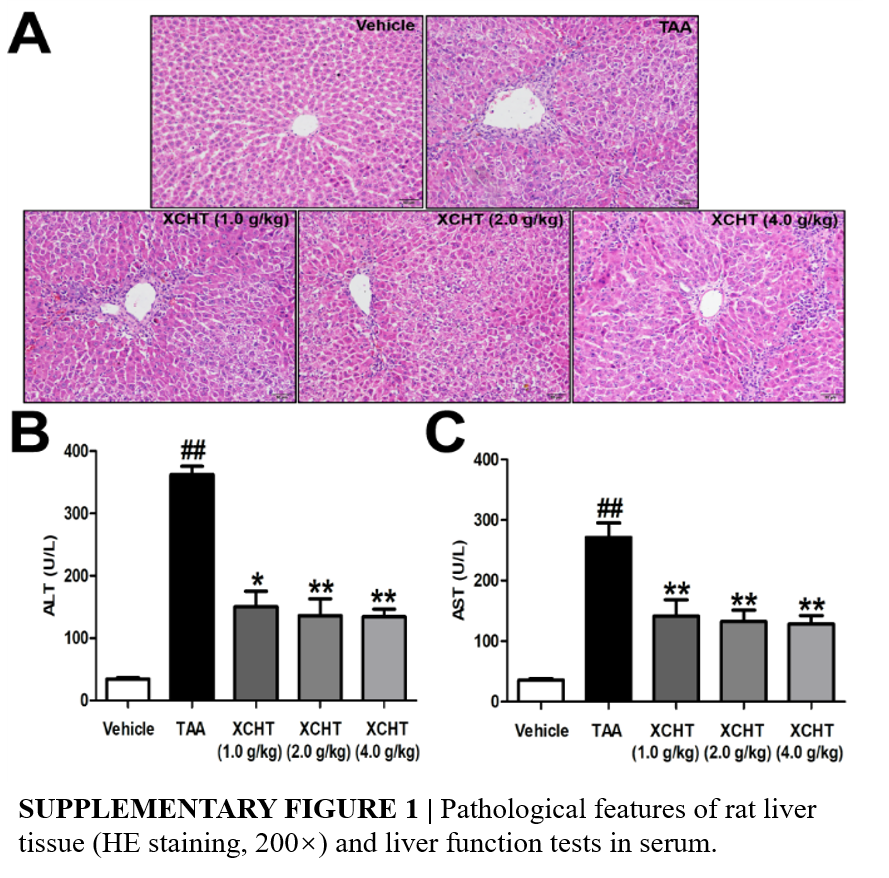

Supplement: Supplementary file 1 [file Image_1.tif]

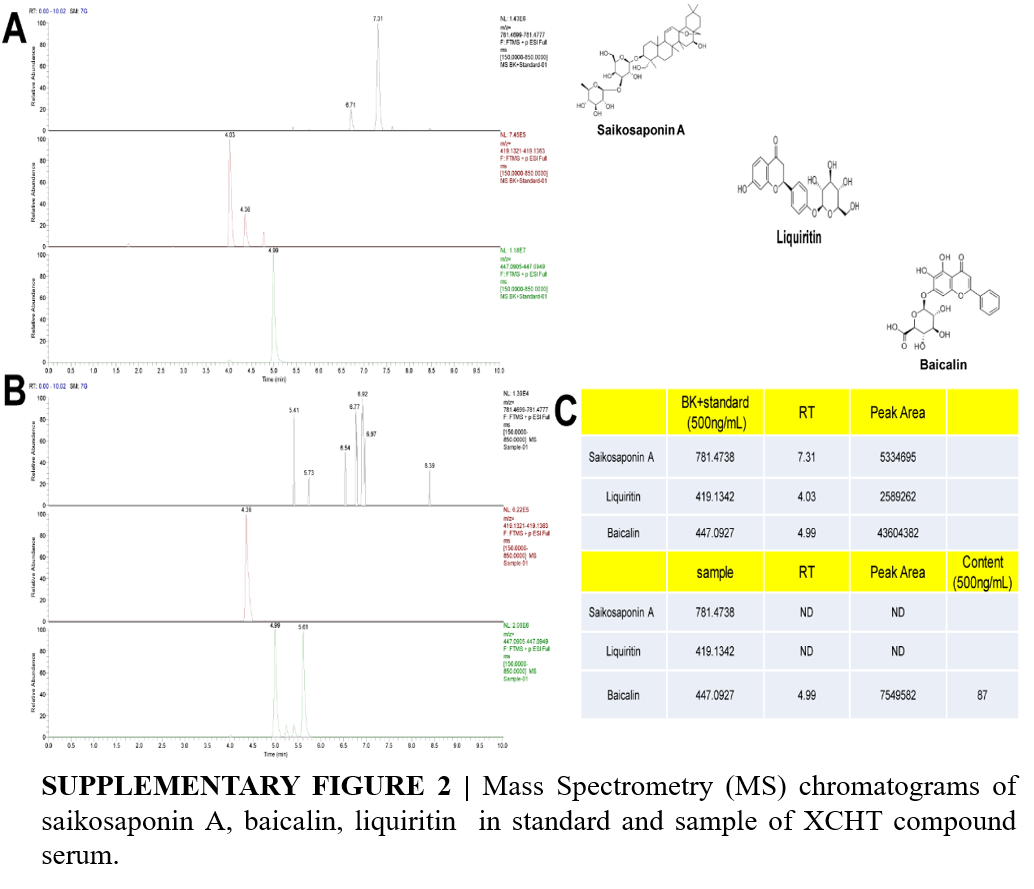

Supplement: Supplementary file 2 [file Image_2.tif]
